# Achieving Ultrahigh Cycling Stability and Extended Potential Window for Supercapacitors through Asymmetric Combination of Conductive Polymer Nanocomposite and Activated Carbon

**DOI:** 10.3390/polym11101678

**Published:** 2019-10-14

**Authors:** Hajera Gul, Anwar-ul-Haq Ali Shah, Salma Bilal

**Affiliations:** 1 National Centre of Excellence in Physical Chemistry, University of Peshawar, Peshawar 25120, Pakistan; hajeragul11@yahoo.com; 2 Institute of Chemical Sciences, University of Peshawar, Peshawar 25120, Pakistan; 3 TU Braunschweig Institute of Energy and Process Systems Engineering, Franz-Liszt-Straße 35, 38106 Braunschweig, Germany

**Keywords:** asymmetric supercapacitor GO@PANI nanocomposite, activated carbon, specific capacitance, coulombic efficiency

## Abstract

Conducting polymers and carbon-based materials such as graphene oxide (GO) and activated carbon (AC) are the most promising capacitive materials, though both offer charge storage through different mechanisms. However, their combination can lead to some unusual results, offering improvement in certain properties in comparison with the individual materials. Cycling stability of supercapacitors devices is often a matter of concern, and extensive research is underway to improve this phenomena of supercapacitive devices. Herein, a high-performance asymmetric supercapacitor device was fabricated using graphene oxide–polyaniline (GO@PANI) nanocomposite as positive electrode and activated carbon (AC) as negative electrode. The device showed 142 F g^−1^ specific capacitance at 1 A g^−1^ current density with capacitance retention of 73.94% at higher current density (10 A g^−1^). Most importantly, the device exhibited very high electrochemical cycling stability. It retained 118.6% specific capacitance of the starting value after 10,000 cycles at 3 Ag^−1^ and with coulombic efficiency of 98.06 %, indicating great potential for practical applications. Very small solution resistance (Rs, 0.640 Ω) and charge transfer resistance (Rct, 0.200 Ω) were observed hinting efficient charge transfer and fast ion diffusion. Due to asymmetric combination, potential window was extended to 1.2 V in aqueous electrolyte, as a result higher energy density (28.5 Wh kg^−1^) and power density of 2503 W kg^−1^ were achieved at the current density 1 Ag^−1^. It also showed an aerial capacitance of 57 mF cm^−2^ at current 3.2 mA cm^−2^. At this current density, its energy density was maximum (0.92 mWh cm^−2^) with power density (10.47 W cm^−2^).

## 1. Introduction

High energy density, long cyclic stability, and rapid charge/discharge capability are the basic requirements for developing electrochemical energy storage devices to be used in hybrid electric vehicles and portable electronics [1]. For example, material with higher specific capacitance are required for supercapacitors (SCs) to be miniaturized for many applications like wearable electronics and on-chip energy storage, etc. Improved device’s energy density is required for supercapacitive (SC) materials, to be incorporated for these applications. For SCs, which operate through physical adsorption of ions, the energy density can be improved by enlarging the active surface area of the material or increasing the operational potential limit. For this purpose, asymmetric devices have been fabricated which utilize the operating potential ranges of two different materials and thus overall potential window is enlarged.

Generally, the active materials that are used for fabrication of SC electrodes are carbon-based materials, transition metal oxides/hydroxides, conductive polymers, and composite materials [2]. Based on charge storage mechanism SCs are of two types [3,4]. In electrical double layer capacitors (EDLCs), the capacitance arises due to electrostatic charge accumulation at the interface of electrode and electrolyte. Carbon-based materials such as graphene, graphene oxide (GO), and activated carbon (AC), show this type of behavior. EDLCs show the advantages of simple process, affordability, good cycle stability, and higher power density [5]. For pseudocapacitors, in which materials such as metal oxides and conducting polymers are used as electrode materials, capacitance arises because of the fast and reversible redox or Faradaic reactions that occurs at electroactive sites at the surface of the electrodes. Pseudocapacitors have the advantages of higher energy density and enhanced specific capacitance [6,7]. Surface area of electrode materials greatly affect performance of both EDLCs and pseudocapacitors. Usually larger surface area is a desired property for achievement of higher capacitance. Suitable pore size with sufficient specific surface area of electrode materials is however, very crucial. For EDLCs the main limitation is lower energy density, while for pseudocapacitive materials the main drawbacks are lower power density and cyclic stability [8]. Best performance can be achieved by combining these two types of materials in a proper way [9].

Nowadays, asymmetric SCs have gained remarkable attention because of their enhanced energy density with maximum power. Mostly, asymmetric SCs are composed of faradaic and non-faradaic constituent electrode materials. Faradaic component is utilized to enhance the energy density while the non-faradaic constituent enhances the power density [10]. Consequently, the overall cell potential window is enlarged because of the combination of both these components [11]. Thus, asymmetric SC that utilizes an aqueous electrolyte is therefore a fascinating approach to assemble a high-performance energy storage device, with its fundamental feature, that is fast charge–discharge, is retained [12].

Among conductive polymers, polyaniline (PANI), polythiophene, and polypyrrole (PPy), and their derivatives have been used for fabrication of electrodes of SCs [13]. PANI has received special attention because of its higher specific capacitance, low cost, easy synthesis, and better stability [14]. However, PANI suffers from low stability due to large volumetric swelling and shrinkage during the charge–discharge process as a consequence of doping and de-doping which results in fast capacitance decline.

In this research work asymmetric device was fabricated using GO and PANI (GO@PANI) nanocomposite doped with DBSA and H_2_SO_4_. Prior to device fabrication, the PANI-GO composite was synthesized through a simple one step polymerization route utilizing two different dopants, i.e.,—H_2_SO_4_ and DBSA, simultaneously. Incorporation of –SO_3_H functional group in GO@PANI nanocomposite lead to hydrogen bonding and π–π delocalization which results in better association among GO and PANI. The internal resistance of the composite was also reduced resulting in ultra-high electrochemical cyclic stability during charge discharge cycles. This composite material was used as positive electrode while AC as negative electrode. The device showed capacitive response in an extended potential window in aqueous electrolyte. To the best of author’s knowledge, there is no report on the fabrication of an asymmetric device with this configuration and high cycling stability.

## 2. Experimental

### 2.1. Materials

Aniline (C_6_H_5_NH_2_), Sigma-Aldrich (Hamburg, Germany), under reduced pressure was double distilled and was then stored in a refrigerator. Chloroform (CHCl_3_), Sulfuric acid (H_2_SO_4_) and Hydrochloric acid (HCl) were purchased from Scharlau Chemie S.A (08181 Sentmenat, Spain) and used as it is. Dodecylbenzenesulfonic acid (DBSA), Ammonium persulfate ((NH_4_)_2_S_2_O_8_)), *N,N*-Dimethylformamide (DMF), Polytetrafluoroethylene (PTFE), and acetone were purchased from Sigma-Aldrich (Hamburg, Germany). All the chemicals were of research grade and used without further purification.

### 2.2. Synthesis of GO@PANI Nanocomposite

Graphene oxide (GO) was synthesized by the modified Hummer’s method [15]. 0.090 g of the synthesized GO was dispersed in distilled water (20 mL) through sonication. Sonication was carried out for 30 min to form homogeneous dispersion. In a round bottom flask, dodecylbenzenesulfonic acid (DBSA) was added to chloroform (50 mL) under continuous stirring. Aniline was then added to the above reaction mixture. After this, 25 mL of 1.1 M of H_2_SO_4_ and 25 mL of 0.09 M ammonium persulfate (APS) were added to it drop wise. GO dispersion was then added slowly to the above reaction mixture and reaction was allowed for 24 h under constant stirring. Green precipitate of GO-polyaniline (GO@PANI) nanocomposite doped with DBSA and H_2_SO_4_ was obtained. After 24 h, the reaction was stopped, washed three times followed by washing with acetone to remove unreacted species. Green precipitate was filtered and dried in oven at 600 °C. PANI was synthesized by the same procedure without adding GO.

### 2.3. Characterization

Samples were characterized through using the following techniques:

Ultraviolet visible (UV–Vis) spectroscopic analysis was accomplished in DMF with the help of Perkin Elmer spectrophotometer (Buckinghamshire, UK) that has a cell of quartz of 1 cm path length. Fourier Transmission Infra-Red (FTIR) spectra were recorded with IR Affinity-S1 (Shimadzu) spectrophotometer (Shimadzu, Tokyo, Japan), scanning over the wavenumber range of 400–4000 cm^−1^, with 2 cm^−1^ resolution. Surface imaging and elemental mapping of the synthesized samples was performed through scanning electron microscopy (SEM) and SEM-Energy Dispersive X-ray (SEM-EDX) analysis (Helios G4 CX Dual Beam microscope equipped with Octane Elite, EFI Berlin Germany).

#### 2.3.1. Electrochemical Characterization

Electrochemical characterization of the newly synthesized nanocomposite was carried out in three electrode assembly. In order to check the real performance of the composites as electrode material for SC application asymmetric devices was fabricated.

#### 2.3.2. Three Electrode Assembly

Electrochemical characterization of composite was conducted in an electrochemical cell utilizing three electrodes assembly with the help of 3000 ZRA potentiostat/galvanostat Gamry (Warminster, PA, USA). 80% composite material, 10% activated carbon, and 10% PTFE were dispersed in DMF and coated on gold sheet (working electrode). A coiled wire of gold was used as counter electrode while saturated calomel electrode (SCE) as reference electrode. 1 M H_2_SO_4_ was used as electrolyte. For PANI and GO@PANI nanocomposite, CVs were accomplished in three electrode setups in potential limit from −0.2–0.8 V at 10 mV/s.

#### 2.3.3. Asymmetric Device

Asymmetric device was fabricated by using two gold sheets as current collectors. The GO@PANI nanocomposite coated electrode was used as positive electrode while AC coated electrode constituted negative electrode. The following equation was used to depict charge storage:

(1) $$\frac{1}{C_{total}}=\frac{1}{C_{+}}+\frac{1}{C_{-}}$$

Here, C_+_ and C_−_ denote the capacitance (C) of the positive and negative electrodes, C_+_ + C_−_ for the whole cell with its coulombic efficiency ~100%, where Q_+_ = m_+_ C_+_ V_+_ and Q_−_ = m_−_ C_−_ V_−_. These are the total charges retained by the positive and negative electrodes, respectively. Where V+ and V− are the corresponding potential limits for positive and negative electrodes. Many factors effect these V+ and V−, which includes many irreversible processes such as electrolyte decomposition and electrode material over-reduction or over oxidation. Asymmetric cell setups are generally based on this equation and is used for its characterization.

Asymmetry exists surprisingly for material that possess pure EDLC behavior. This asymmetric behavior is due to the ion size variation in comparison with pore diameter even in EDL materials. Thus, specific capacitances (Cs) obtained for these two are not identical and for positive (Csp_+_) and negative electrodes (Csp_−_), Csp_−_ ≠ Csp_+_. Consequently, the weights of both electrodes (m_−_ and m_+_) are not identical, in order to get suitable voltage limit for V_−_ and V_+_. In this way dissimilar values of specific capacitance are compensated.

(2) $$m_{-}=\frac{C_{sp+}*V_{+}*m_{+}}{C_{sp-}*V_{-}}$$

The electrochemical characterizations of the assembled device were performed through CV, GCD, and EIS. CV was recorded at various potential limits from zero to 0.6, 0.7, 0.8, 0.9, 1.1, 1.2, and 1.3 V at scan rate of 10 mV/s, in order to choose the optimum potential window. CVs were recorded in the optimum potential window of 0–1.2 V at different scan rate (10, 30, 50, 70, 100, 200, 300, 400, and 500 mV/s). GCD was carried out at potential limit from 0–1.2 V at various current densities (1, 2, 3, 4, 5, 6, 7, 8, 9 and 10 Ag^−1^). Equation (3) and (4) were applied to calculate capacitance and specific capacitance, respectively, where C is capacitance, I is the current applied, Δ*t* time taken by discharge, Δ*V* applied potential limit, Cs is specific capacitance and M is total mass of both electrodes [16]. Energy density (E, Wh kg^−1^) and power density (P, W kg^−1^) of the asymmetric electrode were calculated by applying Equation (5) and (6), respectively. CA is a real capacitance while A is total area of both electrodes. Cyclic stability was checked through repeated cycles at 0–0.7 V potential window for 10,000 cycles.

(3) $$C=\frac{I\times \Delta t}{\Delta V}$$

(4) $$C_{s}=\frac{4\times C}{M}$$

(5) $$E=\frac{1}{2\times 3.6}C\Delta V^{2}$$

(6) $$P=\frac{E_{t}\times 3600}{\Delta t}$$

(7) $$C_{A}=\frac{I\times \Delta t}{A\times \Delta V}$$

To assess material capacitive nature, EIS is an effective technique. Open-circuit potential was employed over a wide range of frequency (f) that is 0.05–105 MHz to collect EIS data. Nyquist plot was drawn for EIS data analysis. Z” (imaginary part of impedance) versus Z’ (real part of impedance) manifest Nyquist plot. From this plot, semicircle diameter expresses a charge transfer resistance. There is also correlation among the imaginary part of impedance |Z| and f. Capacitance was obtained by applying equation C = 1/(2πf|Z|). The linear part of a log |Z| versus log f curve is used for calculation and this plot is known as Bode plot.

## 3. Results and Discussion

### 3.1. Fourier Transform Infrared Spectroscopy

**Figure 1:**
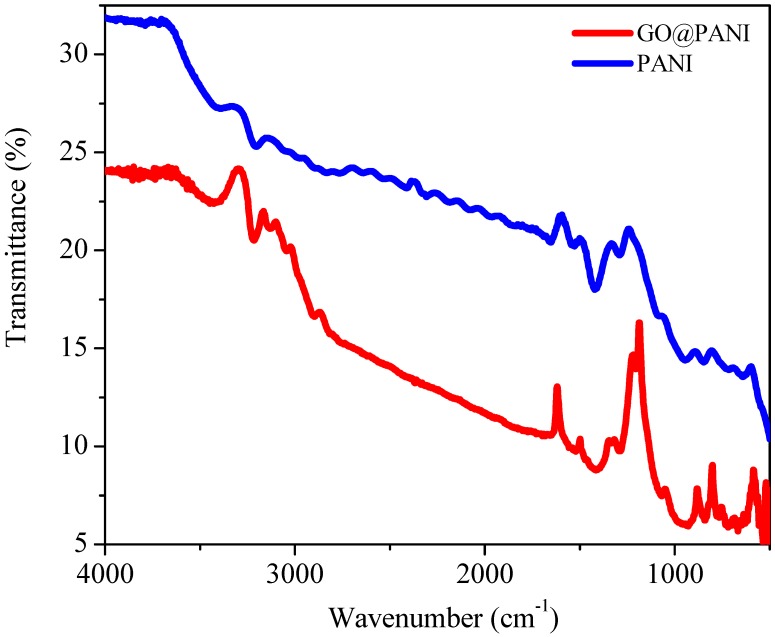
Fourier Transmission Infrared Spectroscopy (FTIR) spectrum of graphene oxide–polyaniline (GO@PANI) nanocomposite and PANI.

The peaks at 3200–3500 cm^−1^ in Fourier Transmission Infrared (FTIR) Spectroscopy represent the N–H stretches of PANI (Figure 1). In the range of 2880–2970 cm^−1^ weak absorption peaks represent the aromatic sp2 hybridized CH stretches of phenyl groups [17]. The peaks at position 1406–1500 cm^−1^ can be assigned to stretching of C=C and C=N on benzoquinone skeleton. C–N stretching of the benzenoid unit show absorption around at 1200 cm^−1^. The 1051 cm^−1^ is assigned to the quinonoid unit of PANI. When correlated with the spectrum of PANI, the spectrum of GO@PANI nanocomposites exhibits an aromatic C–H vibration about at 885 cm^−1^. This peak is the characteristic of ortho-substituted benzene. Thus, it confirms an ortho-GO@PANI formation form aniline molecule via C–N. While shift in characteristic bands of PANI to higher wavenumbers might be attributed to the interlinkage among PANI and GO [18]. For PANI-GO, some characteristic bands of oxygen-containing functionalities are observed illustrating the existence of a conductive composite framework containing both PANI and GO [19].

### 3.2. UV−Visible Spectroscopic Analysis

**Figure 2:**
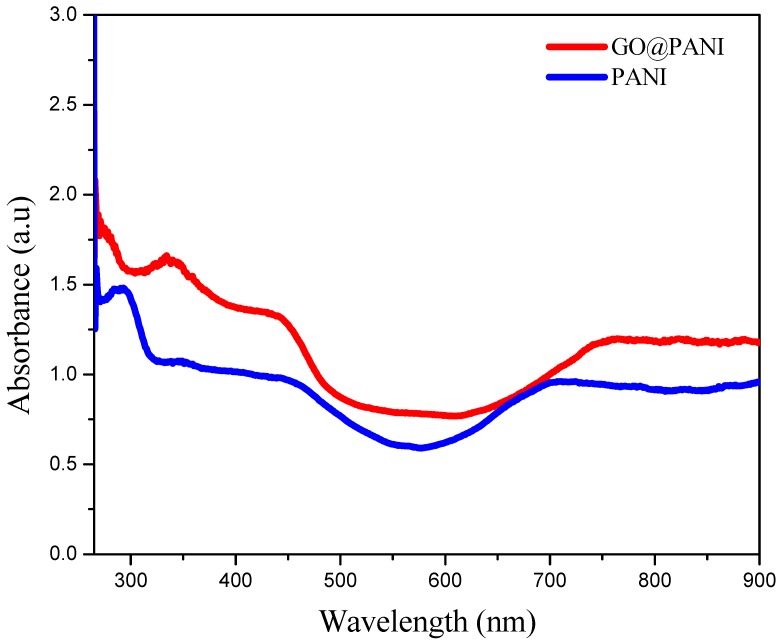
Ultraviolet (UV)-Visible spectra for GO@PANI nanocomposite and PANI.

The peaks at 292 nm in spectrum of the PANI doped with H_2_SO_4_ and DBSA (Figure 2) are because of the π–π* transition of benzene and benzoquinone ring, illustrating that the synthesized PANI is in p-doped state [20]. However, for GO@PANI nanocomposite, the characteristic peak at 339 nm is red-shifted from 292 nm in PANI, which reveal the conjugation between PANI and GO. While the blue-shifting of the band from 466 nm in GO@PANI to 441 nm might be due to the increase in steric hindrance manifesting that PANI and GO are closely connected. UV–Vis spectra along with FTIR results illustrate that PANI in the composite is in the p-doped state that is favorable for the performance of the SC. It can be proposed that GO and PANI combined via electrostatic interactions, π–π stacking and hydrogen bonding.

### 3.3. Scanning Electron Microscopy (SEM) and Energy Dispersive X-ray (EDX) Analysis

**Figure 3:**
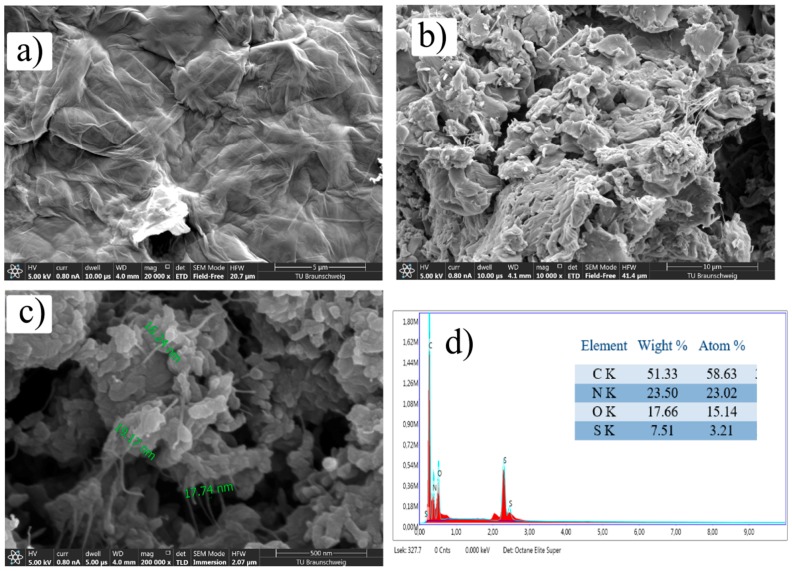
SEM images of (**a**) GO, (**b**) PANI, (**c**) GO@PANI nanocomposite and (**d**) EDX of GO@PANI nanocomposite.

SEM images of GO, PANI, and GO@PANI nanocomposite are given in Figure 3a–c, respectively. GO show typical flake like morphology [21]. From Figure 3b,c, it can be noticed that GO@PANI nanocomposite has a small particle size as compared with PANI. This might be because of the influence of DBSA and GO on the morphology of composites [22,23]. The morphology of this composite material is very different from individual components. The apparent porous structure provides the path for ion transport to inner material [24]. An increase in the overall surface area can consequently lead to formation of more electrochemical active sites of the electrode. The area of contact between the electrolyte solution and the active material increases that is advantageous for the development of the double layer capacitance of the GO component.

In Figure 3d, the EDX analysis of GO@PANI is given. EDX analysis shows the existence of C, O, N, and S elements, witnessing successful incorporation of dopants and GO in PANI backbone.

### 3.4. Electrochemical Study

Cyclic voltammograms (CVs) of PANI and GO@PANI nanocomposite were recorded in three electrode assembly (Figure S1). The area covered by GO@PANI nanocomposite is larger than that of PANI. Overall properties of GO@PANI nanocomposite is better than that of PANI. So, GO@PANI nanocomposite was selected for asymmetric device fabrication. In Figure S2, CVs of activated carbon (AC) and GO@PANI nanocomposite are given and are used for calculation of mass ratio of electrode materials to maintain the voltage equality.

#### Electrochemical Properties of Asymmetric Supercapacitor

##### Cyclic Voltammetry of Asymmetric Supercapacitor Device

To assemble the asymmetric SC device, AC was utilized as the negative electrode while GO@PANI nanocomposite as the positive electrode material. The extent of charge, Q, stored in individual electrodes in an asymmetrical SC must be the identical. The SC cell capacitance relies on voltage splitting across individual electrodes. It primarily relies on mass and specific capacitance of the active material utilized in both electrodes. For symmetric SC the potential window can split symmetrically among the two electrodes by virtue of identical materials used in both electrodes with identical amount of active material. This voltage split is influenced by the capacitance of the active material in individual electrodes. Therefore, to manage the voltage equality, electrode mass was optimized by using the method presented by Snook et al. [25].

**Figure 4:**
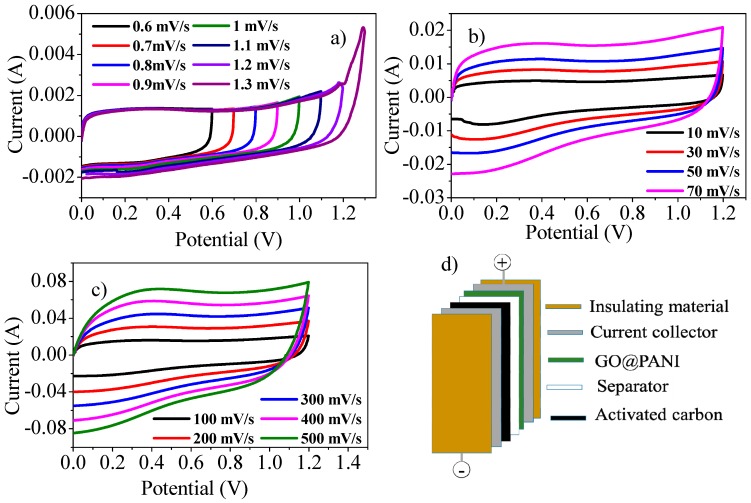
CVs of asymmetric device (**a**) at different potential ranges. (**b**) At various lower scan rates ranging from 10–70 mV/s and (**c**) at numerous higher scan rates ranging from 100–500 mV/s. (**d**) Configuration of asymmetric assembly.

In an asymmetric device, charge storage capacities of the electrodes are not similar. Thus, the capacitance reported corresponds to the full cell. Figure 4a shows CVs of asymmetric SC at various applied potentials at 10 mV/s scan rate. It can be observed that some extra peaks appear at 1.3 V, therefore, 1.2 V was considered as the maximum potential window. Figure 4b show CVs at potential range 0–1.2 V for the asymmetric device at various scan rates. The capacitive response (rectangular shape of the voltammogram) is sustained at increasing scan rates indicating excellent ion diffusion to the inner pores of the composite materials. It also exhibits low contact resistance of electrode materials. The CVs were also recorded by further increasing the scan rate and taking it to the higher values of 500 mV/s (Figure 4c). As the shape was maintained even at these higher scan rates, there is fast ion/electron-transportation between the active material and current collector [26,27,28]. As both of the electrodes have complementary working potential windows, the electrochemical potential limit of the device is extended to 1.2 V. The enlarged device operating voltage limit will positively influence its energy and power density.

This increase in stability of composite materials might be due to the presence of GO in composite. During electrochemical cycling drastic swelling and shrinking of inner particles are prevented by GO sheets. As a result, it gets rid of morphological transformation, enhancing the structural integrity, lifetime, and performance of the material [29].

##### Galvanostatic Charge Discharge (GCD) Analysis of Asymmetric Device

**Figure 5:**
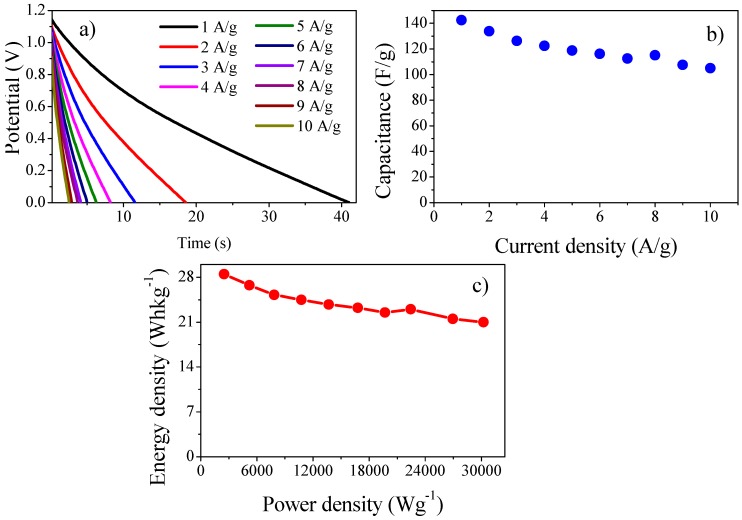
(**a**) Discharge curves of asymmetric devices at different current densities. (**b**) Specific capacitance versus current densities for the asymmetric device. (**c**) The Ragone plot.

**Figure 6:**
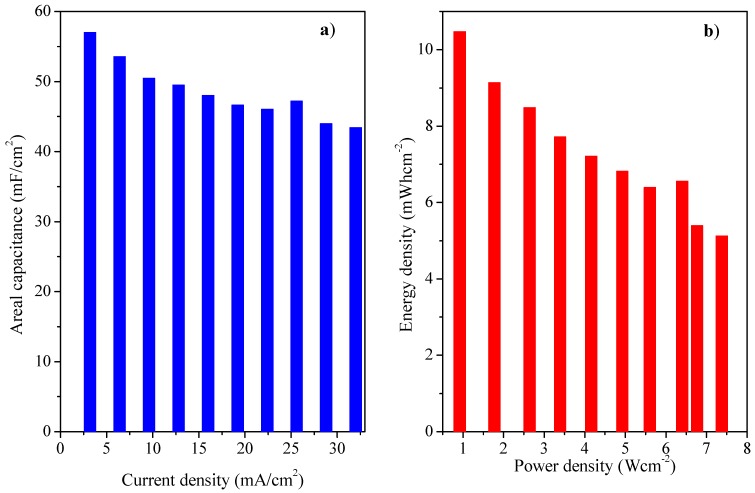
(**a**) Areal capacitance versus current density. (**b**) Ragone plot.

GCD analysis of asymmetric cell was executed in potential limit form 0–1.2 V at current densities from 1–10 Ag^−1^ (Figure 5a). The device showed highest specific capacitance of 142 Fg^−1^ at 1 Ag^−1^ current density. As can be seen from Figure 5b, 73.94% of the capacitance was retained at higher current density 10 Ag^−1^, which shows high sustainability and excellent rate performance [30]. In Figure 5c, the Ragone plot is given. The highest energy density of 28.5 Wh kg^−1^ was achieved with power density of 2503 W kg^−1^ at 1 Ag^−1^ current density [31]. These results reveal superior or comparable performance of the device with other asymmetric devices based on PANI and AC reported before (Table 1). An areal capacitance of 57 mF cm^−2^ was achieved at current density of 3.2 mA cm^−2^ also exhibiting very high energy (0.92 mWh cm^−2^) and power density (10.47 W cm^−2^) (Figure 6a,b). This increase in energy density can be attributed to broadening of the potential window in an aqueous electrolyte due to the asymmetric combination of activated carbon and PANI-GO nanocomposite [32].

##### Electrochemical Impedance Spectroscopic (EIS) Analysis

Electrochemical impedance spectroscopic (EIS) analysis is an excellent tool to explore the interior resistance and the electrochemical performance of electrode material in bulk and at electrode–electrolyte interface [36].

**Figure 7:**
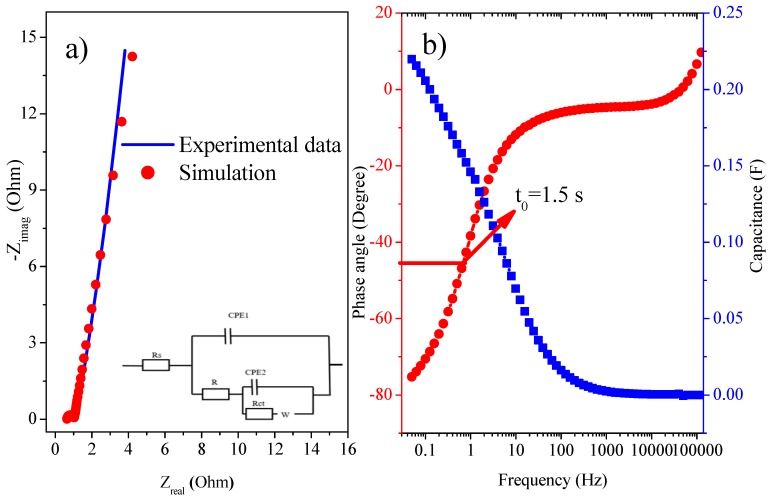
(**a**) Nyquist plot for asymmetric device (Inset: Equivalent circuit model). (**b**) Bode plot of asymmetric SC device.

Impedance test for the device was executed in the frequency range from 0.05 Hz to 100,000 Hz at the open-circuit potential (Figure 7). The Nyquist plot is composed of a depressed semicircle in region of high frequency and about a vertical line in the region of its low frequency (Figure 7a). The small semicircle depicts the favorable electrical conductivity and lower resistance [37].

Equivalent series resistance (ESR), for capacitors is the combination of Rs (solution resistance) and internal resistance (IR) [38]. It can be determined from the Nyquist plot form the point where the semicircle cuts on real axis in the region of high frequency. While charge transfer resistance (Rct) can be determined from radius of the arc in high frequency region.

Introduction of GO in composites leads to faster conduction because ion diffusion pathway is shortened and new electroactive sites are created. Electrical conductivity is also improved due to GO, resulting in low Rct that is helpful for quick charge transfer and enhanced charge storage [39]. As shown in SEM images, these composites possess very porous morphology with nanosized particles. Such texture supports efficient charge transfer, fast ion diffusion, and lower resistance. Material possess fast charge-discharge phenomenon due to very small Rs and Rct, t_0_ values, reduction in values of ESR, and enhanced electrical conductivity of the electrode-electrolyte. These properties make this composite material exceedingly valuable to be used as SC electrode [40].

In the region of low frequency nearly vertical arm of the AC impedance reveal excellent capacitive behavior, which is important for quick diffusion of ions and adsorption in/on the electrode material [39]. This vertical arm at region of low frequency show the capacitive characteristic. Diversion of the slop might be due to pseudocapacitance of PANI present in the composite. This is also cleared from CV and GCD analysis.

In Nyquist plots there is a very small depressed semicircles at the high-frequency region, illustrating the existence of very small Rct. Lower value of ESR, at higher frequency with almost no semicircle and at low frequency about vertical line are because of the high porosity, higher electrical conductivity, and excellent capacitive behavior of electrode material.

At low frequency region, a line diverted from 900 shows diffusion-controlled Warburg behavior [40]. Capacitive behavior is shown by slopping line at low frequencies. Lower resistance values definitely show higher electrical conductivity throughout the entire inner structure. Phase angle dependence on the frequency, knee frequency (*f*_0_) is defined as the phase angle where it attains the value of 450. At knee frequency the capacitive and the resistive impedances are equivalent [41]. Greater resistive characteristic is shown at frequency higher than this knee frequency. Where relaxation time (τ_0_=*f_0_*^−1^) is defined as the smallest time required to discharge whole energy from the device with an efficiency that is higher than 50%. Generally, the higher the knee frequency, the higher the rate capability is (lower t_0_).

Nyquist plots explained by fitting the experimental EIS curve to an equivalent electrical circuit (Figure 7a) shows that equivalent electrical circuit is composed of Rs, Rct, frequency power (n), Warburg (W), resistance of composite film in electrolyte (R), and constant phase element (CPE). Two constant phase elements, CPE1 and CPE2, are applied to replace double layer capacitance and pseudocapacitance, respectively. As, C = CPEn so for n=1, CPE is equivalent to a capacitor [42]. The values of n are in the range from −1 to 1. For n = −1 the CPE is equivalent to an ideal inductor. When n = 0, the CPE is equivalent to an ideal resistor and n = 0.5 stands for diffusion characteristic [43].

From the equivalent electrical circuit value of Rs is 0.640 Ω, R (0.400 Ω), Rct (0.200 Ω), W (1.6 × 10^−6^ S.s^1/2^), CPE1 (0.184 S.s^n^), and CPE2 (0.001 S.s^n^) [43]. Value of n1 is 0.860 and n2 is 0.885. These values are near to one and therefore, illustrate excellent supercapacitive characteristics. A CPE in the equivalent circuit originates from the non-homogeneity, porous nature of the composite film. In the Bode plot (Figure 7 b) a phase angle of −75.27^0^ is observed which is close to −90^0^, illustrating nearly ideal capacitive behavior. At low frequencies, the sloping lines declare the capacitance behavior. This aberration of the straight line from 90^0^ explicate a diffusion-controlled Warburg phenomenon, which is ascribed to the semi-infinite diffusion of protons at the interface of composite with electrolyte.

Additionally, the inhomogeneity or inconsistency in the surface of deposited film as well as the faradaic charge storage might also contribute for this diversion from ideal vertical line in the region of low frequency. From the Bode plot given in Figure 7b, t_0_ at phase angle −45^0^ is 1.5 s. It is an obvious fact that the higher the knee frequency, superior is the rate capability, which results in decreased relaxation time [41].

##### Cycling Stability

**Figure 8:**
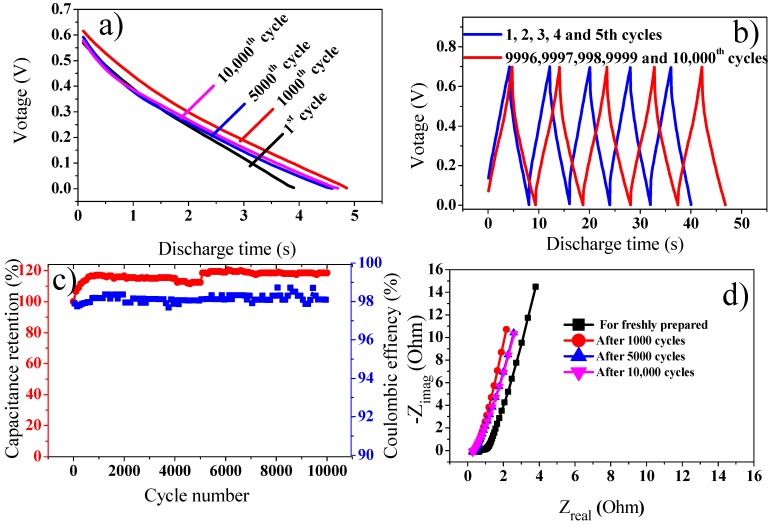
(**a**) Galvanostatic discharge curve for the device at various cycles (1st, 1000th, 5000th and 10,000th). (**b**) Charge discharge behavior of asymmetric device at 1st, 2nd, 3rd, 4th, 5th and 9996th, 9997th, 9998th, 9999th, and 10,000th cycles. (**c**) Cycling stability and coulombic efficiency of the asymmetric device. (**d**) Nyquist plots recorded for freshly prepared, after 1st, 1000th, 5000th, and 10,000th cycles.

The cyclic performance of SC material is a significant indication for its real applications. The device was tested for its cycling stability by applying 10,000 cycles at 3 Ag^−1^ current density (Figure 8a,b).

Interestingly, the specific capacitance showed slight increase and then remains constant. This enhancement in specific capacitance might be due to the insufficient contact of composite material with aqueous electrolyte solution at the commencement of electrochemical process [44]. The initial enhancement of capacitance can perhaps be because of the felicitous wetting of the composite materials with the electrolyte, which promote diminution of the electrode internal resistance [45].

Figure 8c show that GO@PANI nanocomposite retained over 118.6% of the starting value after 10,000 cycles at current density of 3 Ag^−1^ and coulombic efficiency close to 100%. This indicates structural stability of the device [46]. A continuous increase in capacitance can be observed with capacitance retention of 118.6% after 10,000 cycles. This gradual increase in capacitance retention might be ascribed to the activation of material by uninterrupted diffusion of electrolyte inside the open-porous channels of the composite material, which is followed by complete wetting of electrode material [3]. Rise in the number of electroactive sites with the extended charge-discharge cycles are principally responsible for the quick electrochemical redox reactions and hence the cycling stability of the device increases. Moreover, the device also exhibits a superior coulombic efficiency after 10,000 cycles.

This increase in stability can be explained from the EIS study (Figure 8d). Before starting charge discharge cycles EIS was recorded, which show smaller charge transfer and solution resistance. EIS recorded after 1000 cycles depicts further decreased charge transfer resistance and solution resistance, suggesting that the electrode was activated and it becomes more accessible to electrolyte with increasing number of cycles. No degradation of the electrode material was observed even after 10,000 cycles [41,44].

This excellent stability of the device might be attributed to lowered internal resistance, as illustrated by the IR drop in GCD study. The internal resistance devaluation would preclude charge build-up in the composite and smoothens the process of charge injection and extraction [43]. The excellent stability in aqueous acidic electrolytes might be because of the synergistic effect among aligned PANI and the basal planes of GO sheets. In addition, due to the incorporation of –SO_3_H functional groups. Hydrogen bonding and π–π delocalization enable the association among GO and PANI more adequate. Thus, the interfacial resistance among graphene and PANI can be lessened. Thereupon, the electrochemical cyclic stability can be enhanced [45,47].

Table 2, gives a comparison of present work with similar previous study. As can be observed that the asymmetric assembly used in the present study show excellent stability when compared to previous work. This excellent stability might be due to better incorporation of GO in composite material. The GO sheets also prohibit the inner electrode material from drastic swelling and shrinking during the process of electrochemical cycling, thus any morphological changes during extended cycling are eliminated, which results in enhancement of structural integrity, lifetime, and performance of the electrode [29].

**Figure 9:**
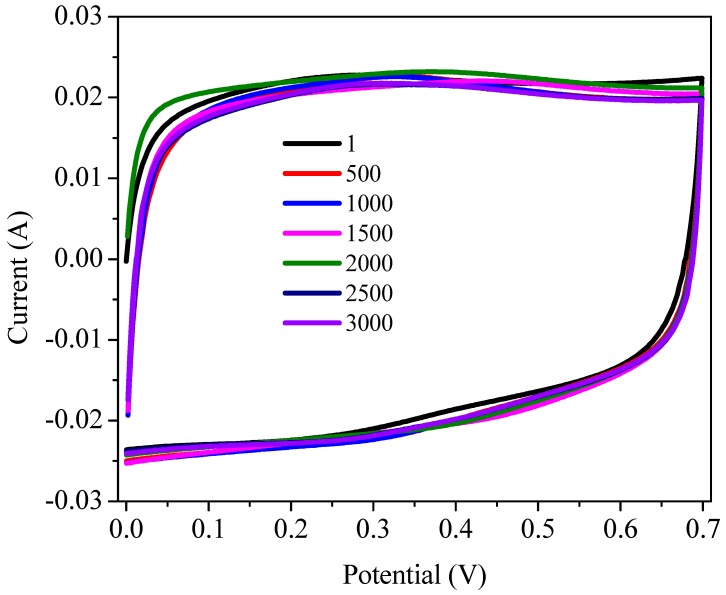
Cyclic voltammograms recorded for the device in potential range of 0–0.7 V at scan rate of 100 mV/s.

Cyclic stability of the device was further confirmed by applying potential cycling in the range of 0–0.7 V at a higher scan rate of 100 mV/s for 3000 cycles. Cycle no one, 500, 1000, 1500, 2000, 2500, and 3000 are given in Figure 9. There is very small change in the area covered by the cyclic voltammogram of one and 3000th cycle with no change in the shape of CV, showing that no degradation takes place. This excellent electrochemical performance of GO@PANI nanocomposite might be due to in situ growth of PANI on GO sheets, which facilitate efficient electron transfer, better use of PANI and reduce the mechanical stress during the process of doping/de-doping. Secondly, as the GO sheets wrap PANI, that can precisely manage the swelling and shrinking of PANI as GO sheets provide new electron transport channels. Additionally, as PANI is embedded in GO framework, thus results in better electrochemical performance as it prevents re-stacking of sheets. Furthermore, interconnected porous structure of PANI and GO facilitate electron transport throughout the whole inner framework of composite due to easy diffusion of electrolytes [48,49].

## 4. Conclusions

The promising properties of PANI and carbon-based material can be combined together to achieve better performance of supercapacitor (SC) devices. The argument is supported by the results obtained in the present work where a highly stable asymmetric SC device with an extended potential window of 1.2 V was fabricated using GO@PANI nanocomposite. The device showed excellent coulombic efficiency (98.06%), ultrahigh electrochemical stability, and specific capacitance retention of 118.6% after 10,000 cycles at 3 Ag^−1^. The device also displayed good capability and retained specific capacitance value of 73.94% at higher current density (10 Ag^−1^). It exhibits its highest energy density (28.5 Wh kg^−1^) with the power density of 2503 W kg^−1^ at 1 Ag^−1^ current density. While it shows an aerial capacitance of 57 m F cm^−2^ at current 3.2 mA cm^−2^. The maximum energy density was 0.92 m Wh cm^−2^ at power density of 10.47 W cm^−2^.

## Figures and Tables

**Table 1:** A performance comparison of specific capacitance values in this work with previous asymmetric SCs.

S. No	Electrode Material	Electrolyte	Potential Window (V)	Current Density/Scan Rate	Specific Capacitance (F/g)	Reference
1	A-PCNFs//PANI PCNFs	1 M L_2_SO_4_	1.8	20 m mV/s	65	[12]
2	sGNS-cMWCNT-PANI//aGNS	1 M H_2_SO_4_	1.6	1 A g^−1^	107	[26]
3	G@MnO2//porous graphene	1 M Na_2_SO_4_	2	0.5 A g^−1^	56	[33]
3	PANI/SG//AC	2 mol/L Perchloric acid	1.4	1 Ag^−1^	128.4	[34]
5	AQ@PNCNTs//PNTs	1 M H_2_SO_4_	1.4	1 A g^−1^	120	[35]
6	GO@PANI //AC	1 M H_2_SO_4_	1.2	1 A g^−1^	142	Present study

**Table 2:** Comparison of cycling stability of present device with previously reported asymmetric devices.

S. No	Positive-Electrode//Negative-Electrode	Electrolyte	Current Density	Rate Capability/Retention	Reference
1	PANI-GO//CPANI-G	1 M H_2_SO_4_	0.5 A g^−1^	90.3% after 5000 cycle	[31]
2	Polyaniline - carbon nanotubes//carbon cloth	0.5M H_2_SO_4_	0.25 Ag^−1^	85% after 1000 cycle	[48]
3	A-PCNFs|| PANI-PCNFs	1M Li_2_SO_4_	2 Ag^−1^	74% after 4500 cycle	[12]
4	GO@PANI nanocomposite //activated carbon	1 M H_2_SO_4_	3 Ag^−1^	118.6 after 10,000 cycle	[Present work]

## Supplementary Materials

The following are available online at https://www.mdpi.com/2073-4360/11/10/1678/s1, Figure S1: CV of PANI and GO@PANI nanocomposite in three electrode setups. Figure S2: CV of AC and GO@PANI nanocomposite in three electrode setups.
