# Supplementary material for: Achieving Ultrahigh Cycling Stability and Extended Potential Window for Supercapacitors through Asymmetric Combination of Conductive Polymer Nanocomposite and Activated Carbon
Source: Polymers (Basel). 2019 Oct 14;11(10):1678. doi: 10.3390/polym11101678 (PMC6835797; doi:10.3390/polym11101678)
Supplement: Supplementary file 1 [file polymers-11-01678-s001.pdf]

A High Performance Asymmetric Supercapacitor Based on Polymeric Nanocomposite with Ultrahigh Electrochemical Stability

Hajera Gul[1], Anwar-ul-Haq Ali Shah*[2] and Salma Bilal[1,3]*

[1] National Centre of Excellence in Physical Chemistry, University of Peshawar, 25120 Peshawar, Pakistan

[2] Institute of Chemical Sciences, University of Peshawar, 25120 Peshawar, Pakistan

[3] TU Braunschweig Institute of Energy and Process Systems Engineering, Franz-Liszt-Straße 35, 38106 Braunschweig, Germany

* Correspondence: s.bilal@tu-braunschweig.de or dresalmabilal@gmail.com (S.B.); Tel.: 0049-531-39163651(S.B) or 0092-919216766 (S.B.) or anwarulhaqalishah@uop.edu.pk 0092-919216652 (A.A.S.)

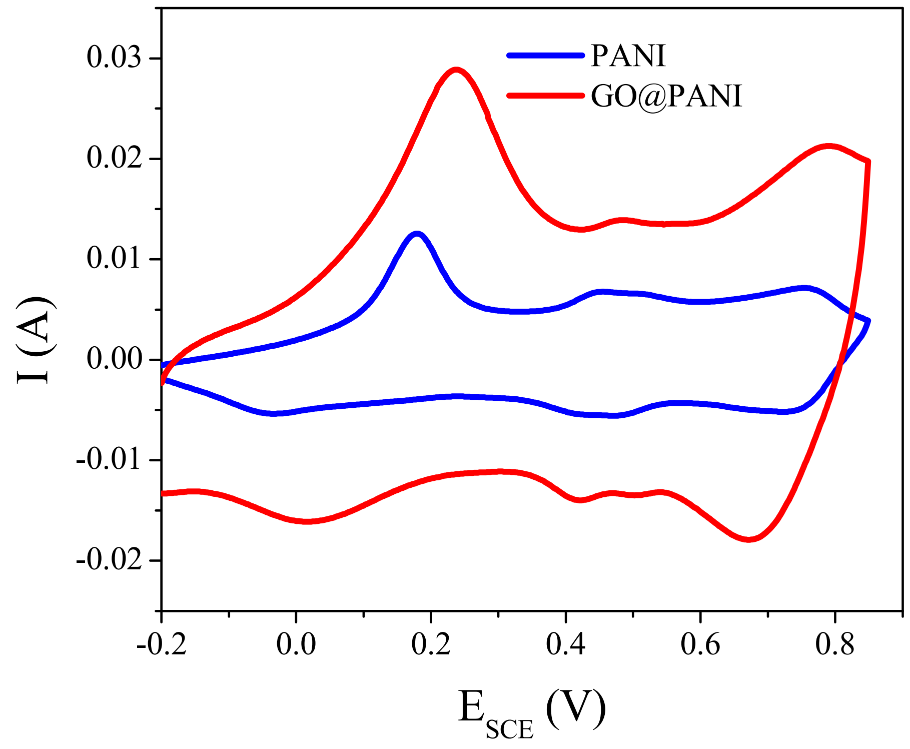

Figure S1: CV of PANI and GO@PANI nanocomposite in three electrode setup.

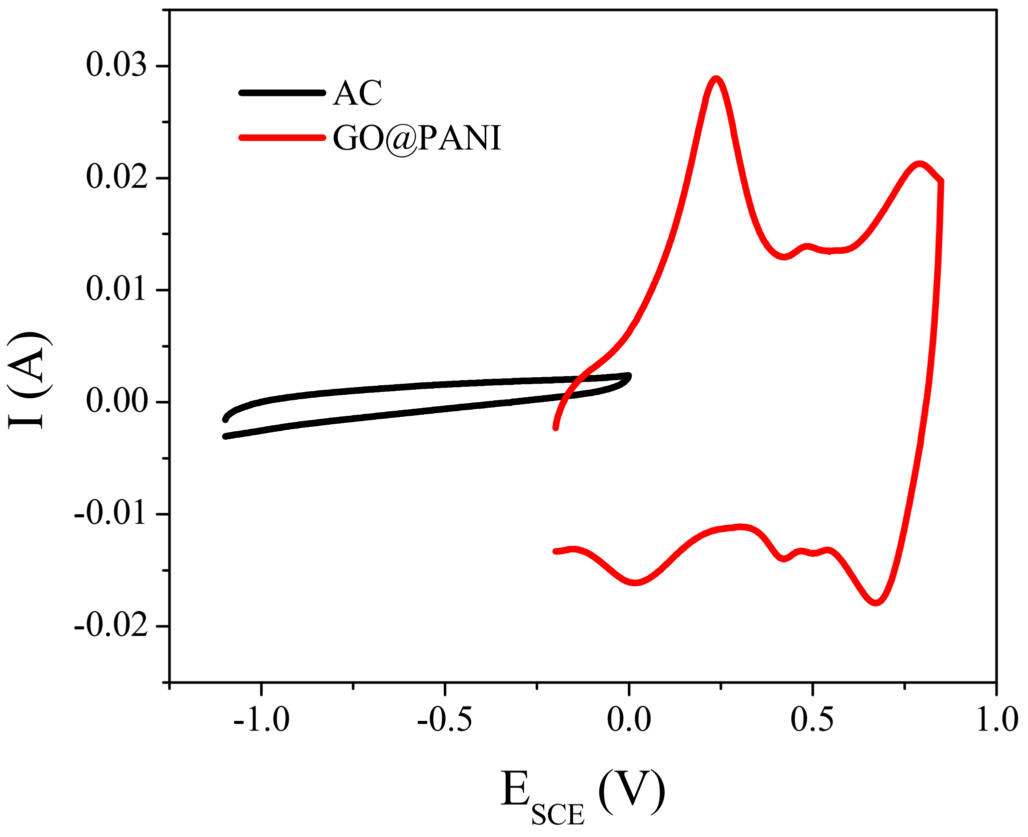

Figure S2: CV of AC and GO@PANI nanocomposite in three electrode setup.
